# Long-term Memory of Sensory Experiences from the First Pregnancy, its Peri-partum and Post-partum in Women with Autism Spectrum Disorders without Intellectual Disabilities: A Retrospective Study

**DOI:** 10.1007/s10803-023-06189-y

**Published:** 2023-11-15

**Authors:** Benedetta Demartini, Veronica Nisticò, Serena Limonta, Vincenza Tarantino, Giulia Stefanelli, Federica Calistro, Laura Giambanco, Raffaella Faggioli, Orsola Gambini, Patrizia Turriziani

**Affiliations:** 1 Unità di Psichiatria 51 e 52, Presidio San Paolo, ASST Santi Paolo e Carlo, Milano, Italy; 2 “Aldo Ravelli” Research Center for Neurotechnology and Experimental Brain Therapeutics, University of Milan, Milano, Italy; 3 Dipartimento di Scienze della Salute, Università degli Studi di Milano, Presidio San Paolo, via A. Di Rudinì, 8, Milano, 20142 Italy; 4 Dipartimento di Psicologia, Università degli Studi di Milano-Bicocca, Milano, Italy; 5 Scuola di Specializzazione in Psichiatria, Università degli Studi di Milano-Bicocca, Milano, Italy; 6 Dipartimento di Scienze Psicologiche, Pedagogiche, dell’Esercizio Fisico e della Formazione, Università degli Studi di Palermo, Palermo, Italy; 7 NeuroTeam Life and Science, Palermo, Italy; 8 U.O.C. Ostetricia e Ginecologia, Presidio Ospedaliero S.Antonio Abate, Trapani, Italy

**Keywords:** Autism Spectrum Disorders, Sensory Sensitivity, Pregnancy, Peri-partum, Post-partum, Motherhood, Gender Medicine

## Abstract

**Purpose:**

To explore the recalled experience of pregnancy and motherhood in women diagnosed with Autism Spectrum Disorders (ASD) without intellectual disabilities, focusing on sensory perceptions and mood.

**Methods:**

We retrospectively evaluated, through an ad-hoc structured interview, the sensory sensitivity during the pre-partum, the peri-partum, and the post-partum of thirty-three mothers with ASD and thirty-two neurotypical mothers. Participants also underwent a psychometric assessment about autistic traits, general sensory sensitivity, and post-partum depressive symptomatology.

**Results:**

Mothers with ASD recalled a higher sensitivity than the comparison group across the three time-points; however, during the peri-partum their recalled hypersensitivity decreases, and in the post-partum it returned as high as before childbirth. The difference in the length of recall between groups did not statistically influence our results. Higher levels of autistic traits correlated with higher depressive post-partum symptomatology.

**Conclusions:**

Mothers with ASD seem to recall their experience of pregnancy, childbirth, and post-partum period differently from neurotypical mothers, particularly in terms of hypersensitivity. The correlation with depressive symptoms and the potential role of oxytocin and of long-term memory (encoding and recollection) are discussed. Further exploring these aspects might give fundamental hints to provide tailored support to mothers with ASD during pregnancy and motherhood.

## Introduction

One of the key diagnostic criteria of Autism Spectrum Disorders (ASD) is the presence of anomalies in the perception and processing of sensory stimuli, in terms of both hyper- and hypo-responsiveness (accounting for, respectively, a lower and a higher sensory threshold for sensory perception) (Crane et al., 2009 DSM-5-TR, 2021). It is estimated that atypical sensory experiences occur in about 90% of individuals with a diagnosis of ASD, both children (Mukherjee, 2017) and adults (Tavassoli & Baron-Cohen, 2012). Moreover, it has been shown that perceptual abnormalities predict the severity of autistic traits, such as the difficulties in social interactions (Mayer, 2017).

In the last decades, the traditional focus on autism in male children has been evolving: there is now a wider acknowledgement that females also exhibit ASD, that children with autism grow into adults with autism (Howlin et al., 2004; Whiteley et al., 2021) and that, of course, women with ASD can also become mothers. However, very little is known about how women with ASD experience pregnancy and motherhood, and on how alterations in sensory perception impact the woman’s well-being (e.g., potential challenges in dealing with changes in their own body, or with the sensory and proprioceptive exchanges that partly characterize the relationship with the newborn). In fact, the research on maternal mental health in the autism field was negatively impacted by the so-called “refrigerator mother theory”, proposed in the mid-20th century, suggesting that autism in children was primarily caused by emotionally distant and cold parenting, particularly by mothers. This theory had significant negative consequences for families of children with ASD: parents, particularly mothers, were wrongly blamed, and severely subjected to shame and stigma; many mothers faced immense emotional distress and social isolation. Over time, this theory has widely been criticized and discredited, and contemporary research (including studies in genetics, neuroimaging, and psychology of development) has firmly established that autism is a complex multi-factorial neuropsychiatric condition. However, this theory has had significant consequences both on women’s wellbeing and on the research on maternal mental health. For example, the fear of being labeled as “refrigerator mothers” might have discouraged mothers from seeking help and/or support for any psychological challenges they faced during pregnancy and motherhood. From a clinical perspective, the focus on maternal mental well-being may have been neglected as, instead, the emphasis was placed on maternal behavior (as a supposed cause of developmental disorders in children). From a societal perspective, the enduring legacy of the harmful notions of the “refrigerator mother theory” may have delayed the broader recognition of maternal mental health during pregnancy and motherhood as a critical aspect of overall well-being. Today, there is a greater recognition of the importance of supporting maternal well-being during pregnancy and motherhood; however, it is crucial to acknowledge that this progress is still ongoing, and challenges related to maternal mental health are still present in many societies. Moreover, within numerous cultures where a persistent misogynistic perspective prevails, a strong anticipation for women to predominantly embody the roles of mothers and wives exists, consequently assigning higher importance to these emotional bonds. In the case of women with ASD, the inherent challenges of the condition may hinder their ability to meet these societal expectations, potentially leading to profound psychological distress and subjecting them to societal prejudices (Rynkiewicz et al., 2018). Hampton et al. (2022) assessed psychopathological symptoms (stress, depression, anxiety) and satisfaction with life in women with and without ASD across several time-points: once during the third trimester of pregnancy, once 2 to 3 months after giving birth, and once 6 months after giving birth. They found that mothers with ASD always scored significantly higher than neurotypical mothers on stress, depression, and anxiety scales although, for both groups, scores for anxiety went down over time. There were no differences between the groups on satisfaction with their life, in their confidence in parenting, and in most areas of parenting style; these results led the author to conclude, on one hand, that parents with autism need proper support during pregnancy and parenthood, and, on the other hand, that neurotypical and neuroatypical parents are equally likely to parent in positive ways, such as being sensitive to their baby’s needs. Hampton et al.’s results about mental health of mothers with ASD are in line with Pohl and colleagues’ (2020) findings, who conducted a comparative study of autistic and non-autistic experiences of motherhood; they found that: (i) mothers with ASD had a higher prevalence of psychiatric comorbidities, including pre- or post-partum depression; (ii) they reported greater difficulties in multi-tasking, in coping with domestic responsibilities and in creating social opportunities for their children; (iii) they reported a greater anxiety and a more frequent feeling of being misunderstood by professionals; (iv) they were more likely to find motherhood an isolating experience, to worry about others judging their parenting, and to feel unable to turn to others for support in parenting. Finally, Grant and colleagues (2022) conducted a systematic review about the experiences of mothers with ASD in infant feeding, and found that, although many women wanted to breastfeed, only a minority of them reported positive breastfeeding occurrences. In particular, the authors highlighted that sensory challenges, pain, and interoceptive differences were one of the main reasons that made breastfeeding impossible, together with unsupportive maternity and infant feeding services and with exhaustion, loss of control over routines and a lack of social support. Overall, this preliminary evidence underscores the significance of further exploring the challenges that neuroatypical mothers might encounter, to ultimately tailor maternity and infant feeding services to address potential specific need. In fact, the choice to embrace pregnancy and motherhood should be a deeply personal decision, and every pregnant woman deserves tailored support that respects her individual needs and circumstances. The results of this study will serve to enhance our understanding of maternity within the context of ASD in the absence of blame and stigma. These insights will be used to design and improve therapeutic approaches that empower and support mothers with ASD throughout their journey into motherhood, ensuring their well-being and the well-being of their children.

### Aim of the Study

The primary objective of this study was to retrospectively explore the experience of pregnancy and motherhood in women diagnosed with ASD without intellectual disabilities. This aim is rooted in the following evidence: (i) the well-documented sensory perception and processing differences observed between individuals with ASD and neurotypical individuals (Crane et al., 2009, DSM-5-TR, 2021); (ii) the suggested connection between perceptual anomalies and challenges in social interactions in individuals with ASD (Mayer, 2017); (iii) the recognition that pregnancy is a period marked by sensory perception and interoception alterations, not only in women with ASD but also in neurotypical ones, as evidenced by previous research (Ruggieri et al., 1979; Nordin et al., 2004; Kwatra et al., 2021). We examined the sensory experiences of mothers with ASD at three specific time-points: during pregnancy, the peri-partum period, and the post-partum period. To investigate the potential specificity of the experience of mothers with ASD, we compared these experiences with those of a group of neurotypical women (comparison group). Additionally, our study aimed to investigate the potential relationship between altered sensory perception and mood, with a particular focus on the post-partum period. Given the aforementioned background, we hypothesized that women with ASD would report a higher sensory sensitivity with respect to the comparison group, and we aimed to explore its trend across the three selected time-points; furthermore, we believed that the potential alteration in sensory sensitivity might have had an influence on women’s mood and well-being during the pregnancy and the post-partum period.

This is a retrospective study: all the participants included, both in the group of women with ASD and in the comparison group, were women who gave birth to their first child up to 15 years before the present research; to maintain clarity and consistency, we limited our investigation to the first pregnancy experienced by each participant in this study.

## Methods

### Participants

Thirty-three women with ASD without intellectual disabilities were recruited at the tertiary level neuropsychiatric clinic of our hospital. Each participant with ASD was diagnosed by a psychiatrist and a psychologist according to DSM-5 criteria (American Psychiatric Association, 2013). To further confirm the diagnosis, all participants underwent Module 4 of the Autism Diagnostic Observation Schedule—2nd version (Hus & Lord, 2014). Exclusion criteria were: (i) age less than 18 years old; (ii) presence of intellectual disabilities (Intelligent Quotient < 90), measured via the Wechsler Adult Intelligence Scale—Fourth Edition (WAIS-IV, Lang et al., 2015), which was administered to participants during the diagnostic assessment; (iii) presence of severe neuropsychiatric comorbidities, that might have influenced their ability to recall information; (iv) inability to understand the instruction of the task, which could have led to incoherent responses to the experimenter’s questions and thus compromised the reliability of our data. Thirty-two neurotypical mothers were recruited from the general population and served as comparison group. Exclusion criteria were: (i) age less than 18 years old; (ii) presence of a diagnosis of ASD or other neurodevelopmental disorders; (iii) presence of any other neurological or psychiatric conditions, that might have influenced their ability to recall information; (iv) presence of intellectual disabilities and/or inability to understand the instruction of the task, which could have led to potentially incoherent responses to the experimenter’s questions and thus compromised the reliability of our data. All participants gave their written informed consent for the study and were free to withdraw from the study at any time without giving further explanation. The study was approved by the local Ethics Committee.

### Procedure

First, participants underwent an interview designed ad-hoc to investigate the participant’s pregnancy and motherhood experience, named “Maternity Questionnaire”. This interview was administered in person, with the experimenter verbally asking questions to each participant. The interview is made of two parts: the first part includes several anamnestic questions, such as each woman’s gynecological and psychiatric history and/or familiarity; the second part investigates participants’ sensory sensitivity during three time-points: pregnancy, peri-partum and three months post-partum, with respect to the pregnancy of their first child. In particular, they were asked to recall their first pregnancy and to describe, on a Likert scale ranging from 1 (not at all) to 7 (completely), their sensitivity towards stimuli across the five sensory domains: smells, other people’s touch, child’s touch, other stimuli’s touch, noise, children or child’s crying, mother’s body changes, tastes, artificial light (strobe, neon, led, etc.), and natural light. A single variable named “Sensory Sensitivity” was created, containing participants’ responses to each question at each time point. The entire questionnaire can be found in Supplementary Materials.

Second, participants completed the following standardized questionnaires: (i) The Edinburgh Postnatal Depression Scale (EPSD, Benvenuti et al., 1999), to evaluate the presence of depressive symptoms after the birth of their first child. (ii) The Sensory Perception Quotient – 35 items (SPQ-35, Tavassoli et al., 2014), a standardized self-report questionnaire to evaluate sensory sensitivity in adults with ASD. (iii) the Ritvo Autism and Asperger Diagnostic Scale Revised (RAADS-R, Ritvo et al., 2011), usually implemented in clinical setting to support the diagnosis of ASD. These questionnaires were administered through a “pencil-and paper” method, and the experimenter remained available to answer any potential questions that each participant might have had.

### Statistical Analyses

Statistical analyses were performed in Statistical Package for Social Science (SPSS), version 27. Significance level was set at α < 0.05, all tests were two-tailed. First, descriptive statistics for sociodemographic and clinical information were calculated for both samples. A variable “Latency” was calculated, representing the years that elapsed between each participant’s first pregnancy and the time of the interview (i.e., Latency = Present Age – Age at first pregnancy).

Second, to analyze changes in recalled sensory sensitivity in the three time-point selected, a Linear Mixed Model (LMM) was implemented with: (i) “Subject” as clustering variable, with a random intercept; (ii) the variable “Sensory Sensitivity” as dependent variable; (iii) “Latency” as a covariate; (iv) “Group” (comparison group vs. group of mothers with ASD) and “Time” (pre-partum vs. peri-partum vs. post-partum) as independent variables, with respect to which the fixed effects and the interaction effect were calculated; pairwise comparison were calculated applying Bonferroni correction for multiple comparison. Moreover, three additional LMMs were run to properly investigate whether the different times in life when our participants experienced their first pregnancy had an actual effect on our data: hence, we inserted, separately, the Age at the time of the interview, the Age at first pregnancy, and the Age at last pregnancy as covariate. Results are reported in the supplementary materials.

Finally, a series of t-test for independent sample was run to assess the presence of differences between mothers with ASD and neurotypical mothers at the psychometric variables (RAADS-R, SPQ-SF35, and EPDS); t-test results are reported according to Levene’s test for homogeneity of variance; according to Bonferroni correction for multiple comparisons, differences between groups were considered significant when p < 0.004 (p = 0.05 / 12 comparisons, consisting in the RAADS-R, SPQ-SF35, and EPDS Total Scores and subscales). Finally, to assess potential associations between sensory sensitivity and autistic traits and post-partum bonding between mother and child, non-parametric Spearman’s correlations were performed between questionnaires’ scores, in the two groups separately.

## Results

### Sociodemographic and Clinical Information

The mean age of participants was 45.4 ± 6.9 years (range = 30–63) for the group of mothers with ASD and 40 ± 8.6 years (range = 29–57) for the comparison group. The mothers with ASD had a mean number of 1.93 children (SD = 0.74, range = 1–4), whereas mothers in the comparison group had a mean number of 1.37 children (SD = 0.49, range = 1–2). With respect to the group of mothers with ASD, their mean total Intelligence Quotient was 117.72 ± 10.3 (range = 101–140) and their mean Autism Diagnostic Observation Schedule score was 9.88 ± 2.83 (range = 7–17). There were no differences between groups with respect to the average age of their first pregnancy, which was investigated in the present study (group of mothers with ASD: 30.4 ± 4.8 years; comparison group: 32 ± 4.4 years) and of their last pregnancy (group of mothers with ASD: 34.3 ± 4.8 years; comparison group: 33.3 ± 4.9 years), (all p > 0.05). However, there was a significant difference with respect to the Latency, i.e., the average years that elapsed between their first pregnancy and the present interview (group of mothers with ASD: 15 ± 8.7 years; comparison group: 7.9 ± 7.8, t = -3.483, p < 0.001). The sociodemographic characteristics of the samples and clinical records related to the birth of the first child are reported in Table 1.

**Table 1:** Socio-demographic characteristics and clinical records

	ASD (n = 33)	Comparison (n = 32)	Statistics	*p*
Socio-demographics				
Age (years)	45.4 (6.9, 30–63)	40 (8.6, 29–57)	*t* = 2.87	0.006
Education (Median)	Bachelor’s degree (16 years)	Bachelor’s degree (16 years)	Χ^2^ = 0.373	0.541
Job (Unemployed / Employed / Freelance)	26/1/6	25/2/5	Χ^2^ = 0.429	0.807
Children	1.94 (0.75, 1–4)	1.37 (0.49, 1–2)	*t* = 3.58	0.001
At least a child with ASD	22	0		< 0.001
At least a child with other neurodevelopmental disorders	8	0		0.005
Psychiatric family per ASD	7	2		0.149
General psychiatric family	17	5		0.004
Age at first pregnancy	30.4 (4.8, 17–47)	32 (4.4, 26–42)	*t* = 1.233	0.222
Age at last pregnancy	34.3 (4.8)	33.3 (4.9)	*t* = -0.868	0.388
Latency (i.e., years of recall)	15 (8.7)	7.9 (7.8)	*t* = -3.483	< 0.001
First pregnancy				
Gestational weeks	38.7 (3.4, 24–42)	39.6 (1.5, 35–42)	*t* = 1.3	0.199
Pregnancy plan:			Χ^2^ = 8.94	0.011
Planned	22	28		
Not- planned	11	2		
Medically assisted procreation	o	2		
Type of partum:			Χ^2^ = 5.51	0.238
Vaginal	16	13		
Induced	8	4		
Planned Caesarean section	3	4		
After induction Caesarean Section	2	8		
In emergency Caesarean section	4	3		
Epidural analgesia	25	14		0.02
Neonatal Intensive Care Unit	2	3		0.999
Breastfeeding type (Exclusive / Formula /Mixed)	18 /8/7	20/4/8	Χ^2^ = 1.49	0.475
Breastfeeding duration (Median)	6–12 months	3–6 months	Χ^2^ = 0.248	0.619
BMI gain with pregnancy	4.85	5.38	*t* = 0.833	0.409
Previous abortion	4	9		0.12
Ritvo Autism Asperger Diagnostic Scale (RAADS-R)				
Total score	150.6 (40.4)	35.8 (23.1)	13.9	< 0.001
Social Relatedness	71.1 (18.9)	15.4 (11.5)	14.1	< 0.001
Circumscribed interests	30.7 (8.2)	9.3 (6.1)	11.7	< 0.001
Language	11.5 (3.8)	2.7 (2.3)	11.1	< 0.001
Sensory motor	40 (12.8)	8.6 (7.7)	11.8	< 0.001
Sensory Perception Questionnaire (SPQ-SF35)				
Total score	38.4 (14.3)	55.4 (13.7)	− 4.88	< 0.001
Vision	6.5 (3.8)	11.1 (2.9)	− 5.34	< 0.001
Hearing	6.5 (2.8)	10.7 (2.4)	− 6.5	< 0.001
Touch	8.3 (4.8)	15 (4.1)	− 6	< 0.001
Smell	10.9 (4.9)	13.4 (4.3)	− 2.2	0.030*
Taste	3.3 (2.1)	5.1 (2.6)	-3	0.002
Edinburgh Postnatal Depression Scale (EPSD)				
Total Score	11.2 (14.2)	8.5 (6.5)	1.6	0.117

In parentheses are standard deviation and range

* Bonferroni correction for multiple comparison, applied to the series of t-test: p = 0.05 / 12 = 0.004; hence, the difference between group at the SPQ-SF35 Subscale Smell cannot be considered statistically significant

### The Maternity Questionnaire on Sensory Sensitivity

**Fig. 1:**
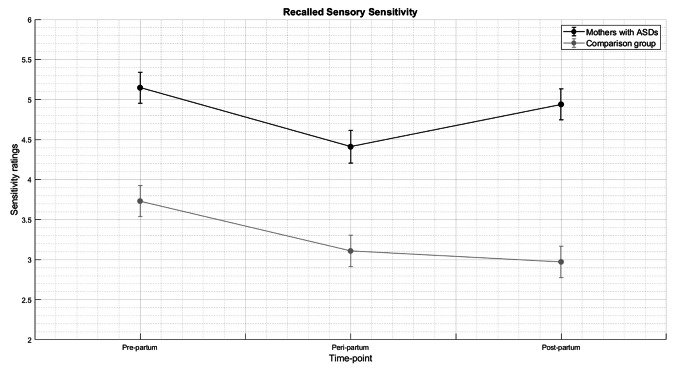
Recalled sensory sensitivity during the pre-partum, peri-partum, and post-partum

A significant interaction effect between “Group” and “Time” emerged (F (2, 1779.58) = 4.895, p = 0.008). Women with ASD reported a significantly higher Sensory Sensitivity than neurotypical women across all the three time-points (with a main effect of group (F (1, 61.54) = 37.681, p < 0.001) and a main effect of time (F (2, 1779.545) = 18.827, p < 0.001). Pairwise comparison showed that, within the comparison group, sensitivity significantly decreased from pregnancy to peripartum (p < 0.001) and remained lower during the post-partum (p = 1); within the group of mothers with ASD, sensitivity significantly decreased from pregnancy to peripartum (p < 0.001) as well but returned higher during the post-partum (p = 0.538). (Fig. 1). Latency did not have a significant main effect (F (1, 60.947) = 1.627, p = 0.207), hence the difference between groups in the years that passed from their first pregnancy and the time of the interview did not influence our results in the reported sensory sensitivity. Further statistical details are reported in Table 2. Furthermore, also the age at the time of the interview, the age at the time of the first pregnancy, and the age at the time of the last pregnancy did not have a significant effect on our data (all p > 0.05, Supplementary Materials).

**Table 2:** Results of the Linear Mixed Model on the Maternity Questionnaire Abbreviation: ASD = Group with mothers with ASD; C.I. = Confidence Interval; Comp = Comparison group; Df = Degrees of freedom; EMM = Estimated Marginal Means; LMM = Linear Mixed Model; SE = Standard Error

**LMM Estimated marginal means**							
**Group**	**Time-point**	EMM*	**SE**	**Df**	**95% C.I., Inf. Lim.**	**95% C.I., Sup. Lim.**	
*Comp*	*Pre-partum*	3.732	0.195	97.774	3.345	4.118	
	*Peri-partum*	3.109	0.195	97.594	2.723	3.496	
	*Post-partum*	2.973	0.196	99.681	2.584	3.361	
*ASD*	*Pre-partum*	5.148	0.193	98.037	4.766	5.531	
	*Peri-partum*	4.411	0.206	125.001	4.004	4.818	
	*Post-partum*	4.940	0.193	97.781	4.557	5.322	
*Covariate assessed for Latency = 11.4059							
Main effects							
	**Error**	**F**	**p**				
*Intercept*	1, 61.298	364.221	< 0.001				
*Group*	1, 61.540	37.681	< 0.001				
*Time*	2, 1779.545	18.827	< 0.001				
*Latency*	1, 60.947	1.627	0.207				
Group * Time	2, 1779.580	4.895	< 0.008				
Pairwise comparisons within time-points							
Timepoint	**Group comparison**	**EMM difference**	**SE**	**Df**	p**	**95% C.I., Inf. Lim.**	**95% C.I., Sup. Lim.**
*Pre*	*Comp - ASD*	-1.416	0.283	94.614	< 0.001	-1.979	-0.854
*Peri*	*Comp - ASD*	-1.302	0.292	106.638	< 0.001	-1.881	-0.722
*Post*	*Comp - ASD*	-1.967	0.284	95.426	< 0.001	-2.531	-1.403
**Bonferroni-corrected for multiple comparisons							
Pairwise comparisons within groups							
Group	**Timepoint comparison**	**EMM difference**	**SE**	**Df**	p**	**95% C.I., Inf. Lim.**	**95% C.I., Sup. Lim.**
*Comp*	*Pre - Peri*	0.623	0.157	1773.151	< 0.001	0.247	0.999
	*Pre - Post*	0.759	0.158	1776.170	< 0.001	0.380	1.138
	*Peri - Post*	0.136	0.158	1776.125	1	-0.243	0.515
*ASD*	*Pre - Peri*	0.738	0.171	1788.193	< 0.001	0.329	1.146
	*Pre - Post*	0.209	0.155	1773.431	0.538	-0.164	0.581
	*Peri - Post*	-0.529	0.170	1787.940	0006	-0.937	-0.121

**Bonferroni-corrected for multiple comparisons

### Psychometric Assessment and Correlational Analyses

As expected, mothers with ASDs scored significantly higher than neurotypical mothers at the RAADS-R (Total scores and subscales, all p < 0.001). Consistently with the results at the Maternity Questionnaire, mothers with ASD obtained significantly lower scores in the SPQ-SF35 than the comparison group both at the Total Scores and at the subscales Vision, Hearing, Touch, and Taste (indicating a higher hypersensitivity to stimuli, all p < 0.004, corrected for multiple comparisons). Finally, no difference between groups emerged at the EPDS (p = 0.117 – see Table 1 for further results). In the group of mothers with ASD, a positive correlation was found between the EPSD score and the RAADS-R score (rho = 0.569, p = 0.002), which was not significant in the comparison group (rho = 0.04, p = 0.826), suggesting that the severity of autistic traits was associated with the severity of post-partum depressive symptomatology.

## Discussion

The aim of the present study was to retrospectively investigate the experience of pregnancy and motherhood in women diagnosed with ASD without intellectual disabilities. The results emerging from this investigation are aimed to enhance our comprehension of maternity within the context of ASD, in order to design tailored therapeutic approaches that support mothers with ASD throughout their journey into motherhood, ensuring their well-being.

We assessed the recollections of a group of mothers with ASD and a group of neurotypical mothers (comparison group) about the sensory perception that they experienced during the pregnancy of their first child, focusing on three specific time-points: the pre-partum, the peri-partum, and the post-partum; we also investigated their memories about their mood, with a specific focus on depressive symptoms of the post-partum period.

First, we found that women with ASD recalled an averagely higher sensory sensitivity than women in the comparison group, across the three time-points retrospectively assessed. This was demonstrated both by higher scores on the Maternity Questionnaire and by lower scores at the SPQ-SF35, which indicates that women with ASD reported a lower sensory threshold (and thus a higher sensory sensitivity) than the comparison group. This result is not surprising: it is already well-known in the literature (Tavassoli & Baron-Cohen, 2012) and represented in the DSM-5-TR criteria for ASD diagnosis (American Psychiatric Association, 2021) that adults with ASD have a hyper-responsiveness to external sensory stimuli compared to neurotypical adults. Moreover, the literature on gender differences with respect to this topic is growing: Grant and colleagues (2022) conducted an observational study on a group of ASD individuals investigating the incidence of the Central Sensitivity Syndrome (CSS - a group of related conditions that are thought to include an underlying sensitization of the central nervous system, in which heightened sensory sensitivity is a common feature): they found this diagnosis to be very common in people with ASD and, in particular, that women with ASD were more likely to report a CSS diagnosis and to experience more CSS symptoms, with respect to men with ASD. However, the research about the experience of pregnant women with ASD, with a particular focus on sensory sensitivity and issues related to body changes, is still in its infancy: it has been shown that sensory challenges contributed to discomfort, distress, or anxiety in many women with ASD, and in some cases had a significant impact on the individuals’ ability to communicate with others (Rogers et al., 2017; Samuel et al., 2022; Talcer et al., 2023). In this study, we made a step forward by finding that the trend of recalled sensory sensitivity levels across the three time-points assessed differs between neurotypical and neuroatypical women. In particular, in our comparison group, sensory sensitivity was reported as significantly higher during pregnancy with respect to peripartum and post-partum: this result is in line with previous studies showing that changes in sensory perception during pregnancy in neurotypical women are commonly experienced (an abnormal smell and/or taste perception (Nordin et al., 2004), a modification in cutaneous sensitivity (Ruggieri et al., 1979), and in hearing threshold levels (Kwatra et al., 2021) were reported). On the other hand, within the group of mothers with ASD, sensitivity is recalled as significantly decreased from pregnancy to peripartum (seemingly getting closer to the levels reported by neurotypical women) but, in the post-partum period, it is reported as high as during pregnancy (Fig. 1). Hence, further considerations on both the moment of childbirth and its long-term recollection are needed.

Although it varies for each woman, the experience of delivery is generally painful due to uterine contractions, which are mediated by the release of oxytocin, a key hormone in labor and lactation. It might be speculated that the lower sensory sensitivity that we found during the peri-partum might be associated with the release of oxytocin itself. The role of oxytocin in ASD has been widely studied in the last decade: Quattrocki and Friston (2014), in a comprehensive review, underlined that it seems to play a role in sensory attenuation (including proprioceptive and interoceptive signals), and suggested that people with ASD might suffer of a developmental neuromodulatory deficit in oxytocin. Moreover, thanks to its to receptors distributed in various brain regions (including the limbic system), oxytocin also works as a neurotransmitter, involved in the regulation of emotions (Huber, 2005), and acts as a mediator of several social behaviours involved in forming attachments, ultimately being branded as a “social hormone”. For this reason, this neurotransmitter has been also suggested as a possible therapy to improve social performances in individuals with ASD without intellectual disabilities (Hollander et al., 2007; Andari et al., 2010; Anagnostu et al., 2012; Watanabe et al., 2015; Yamasue, 2016; Demartini et al., 2021). Our results might be interpreted in this framework, and we could speculate that oxytocin plays a role in modulating (and, more specifically, decreasing) the hypersensitivity typical of people with ASD, ultimately determining a better well-being of the individual and therefore a greater predisposition to social interaction.

With respect to the role of memory in our investigation, several studies recently assessed neurotypical women’s recollections of their childbirth experiences, up to five years postpartum. Takehara et al. (2014) conducted a prospective cohort study assessing the childbirth experience of 1168 women in Japan at a few days postpartum and 5 years later; they concluded that, averagely, women still remember their childbirth experience clearly at 5 years after the childbirth. On the other hand, Norvell et al. (1987) questioned the reliability and validity of the retrospective assessment of labor pain, that we did not directly assess in our study. Although it is a grueling experience for mothers, it seems that, in recall, labor pain is underreported; in fact, Waldenström et al. (2009) demonstrated that neurotypical Swedish women show a significant decrease in the ratings of their labor pain when investigated 2 months, 1 year, and 5 years after childbirth. The authors concluded that in-labor pain and long-term memory of pain should be discussed as two separate outcomes, involving different memory systems. Again, according to Farley et al. (2019) this could be due to oxytocin, when physiologically released at the time of birth since its action on the central nucleus of the amygdala can modulate the memory of pain in a more favorable light. It could therefore be hypothesized that this mechanism also acts on the hypersensitivity of autistic women during childbirth: exactly as for pain, the release of oxytocin could decrease its intensity in memory; hence, our results would not represent an actual decrease in hypersensitivity, but only an underestimation of their intensity in women’s memory (which, indeed, occurs also in our comparison group). Overall, while it is possible that autistic mothers have a different moment-by-moment experience with childbirth and the post-partum period, it cannot be excluded the possibility that they have different ways of encoding and storing long-term memories of those experiences. Moreover, our results can be attributed to various environmental, cognitive, and psychological factors: given the increased attention towards one’s own body during pregnancy, mothers may have reported a heightened recall of sensitivity pre-partum; given the intense nature of labor, the recollection of sensitivity to external stimuli might be less vivid due to their lower low salience; finally, the changes in each own’s routine during the post-partum might have had a different impact on neurotypical and neuroatypical mother, ultimately influencing their experience and their recall of sensory perception. Hence, further studies are needed to better understand the potential mechanism behind our findings.

Finally, we found that mothers with ASD scored significantly higher than neurotypical mothers at the RAADS-R (confirming the ASD diagnosis) and lower at the SPQ-SF35 (confirming their hypersensitivity). Through correlational analysis, we found that the presence of higher autistic traits was associated with a higher symptomatology suggestive of post-partum depressed mood. These findings provide additional evidence supporting the necessity of investing in the development of targeted therapeutic approaches aimed at offering a significant and ongoing support to mothers with ASD during pregnancy and motherhood overall, with proper attention to the mothers’ wellbeing.

## Limitations, Future Perspectives, and Conclusions

The main limitation of this study lays in its retrospective nature: the entire assessment relied on participants’ self-reported long-term memories, and not on a direct evaluation of sensory perceptions during pregnancy and the post-partum period.

Furthermore, mothers with ASD had, on average, a higher age than those in the comparison group. As a result, their memories of their first child extended further back in time, as indicated by the variable “Latency”. It’s worth noting that while this age difference was statistically significant, it was relatively small, amounting to only five years; most importantly, this difference in the length of recall did not have a statistically significant impact on our results. Moreover, we focused only on the first pregnancy, and it must be noted that the recall of potential subsequent pregnancies (that not all women in our sample experienced) might have influenced our results. We did not examine the experience of breastfeeding, which is known to significantly affect women’s well-being and their bonding with the newborn: hence, it should be investigated in a future study. Of note, the majority of the women in our sample in both groups were unemployed, and we did not investigate whether it was a personal choice, or if it was dictated by potential difficulties in finding a job; this might have had an impact on the mood of each participant at the time of the interview. Finally, it should be noted that the “Maternity Questionnaire” was designed specifically for the study, thus, it lacks statistical validation and normative data.

Future prospective research, recruiting pregnant women before childbirth and tracking their ratings about sensory sensitivity and mood for up to five years postpartum, is firmly needed to gain a better understanding of women’s experiences, and to determine whether our results stem from the initial experiences themselves or from how those early experiences are encoded as long-term memories.

In conclusion, mothers with ASD seem to recall the sensory experience during pregnancy, childbirth, and the post-partum period differently from neurotypical mothers, particularly in terms of hypersensitivity, although during the peri-partum the hypersensitivity is recalled as decreased. Our results might help in designing specific therapeutic approaches to provide a proper support to mothers with ASD during pregnancy and motherhood overall, and to build awareness (both in the medical staff and in the general population) about the enhanced efforts that women with ASD might experience in this delicate period of life.
